# CHANGING THE CULTURE OF ADVANCE CARE PLANNING, DEATH, DYING, AND BEREAVEMENT CARE IN NURSING HOMES

**DOI:** 10.1093/geroni/igz038.645

**Published:** 2019-11-08

**Authors:** Toni Miles, Amanda Brown, Pamela O’Rouke

**Affiliations:** 1 University of Georgia College of Public Health, Athens, Georgia, United States; 2 CHSGa, Gray, Georgia, United States

## Abstract

Cultural change, using an Eden Alternative metaphor, means that palliative care becomes ‘the servant of genuine human caring, not its master’. This project, funded through the Georgia Civil Monetary Penalty Fund Reinvestment Program (CMPRP), aimed to gather information from multiple stakeholders to promote changes in the culture of advance care planning, death, dying, and bereavement care in nursing homes. Staff, residents and families from nine nursing homes provided perspectives through qualitative interviews (n=70). Participants were drawn from both small facilities and corporate entities with multiple sites, and included residents in both short and long-stay beds. Qualitative analysis of interview data resulted in the publication and dissemination of Best Practice in Bereavement Care Guides, with separate versions for residents, families, and staff. This presentation will describe this project in terms of the process of consensuses building and obtaining funding, as well as development of the guides through qualitative interview data.

